# The Influence of Self-Perception on Manipulative Dexterity in Adults with Multiple Sclerosis

**DOI:** 10.1155/2021/5583063

**Published:** 2021-08-16

**Authors:** Rosa M. Martínez-Piédrola, Cristina García-Bravo, Elisabet Huertas-Hoyas, Patricia Sánchez-Herrera Baeza, Jorge Pérez-Corrales, Carlos Sánchez-Camarero, Marta Pérez-de-Heredia-Torres

**Affiliations:** Department of Physical Therapy, Occupational Therapy, Rehabilitation and Physical Medicine, Faculty of Health Sciences, Rey Juan Carlos University, Madrid, Spain

## Abstract

**Background:**

Multiple sclerosis is a disorder which causes a loss of functionality, affecting the person's ability to perform activities of daily living, such as interpersonal interactions and relationship, dressing, self-care, or bathing, as well as having a negative impact on work and leisure activities.

**Aims:**

This study examined the relationship (correlational or associations/predictive) between self-perceived quality of life and performance of manipulative dexterity. Also, this study sought to measure predictors of dexterity. *Study Design*. A cross-sectional study from two associations of MS within the Community of Madrid, Spain. *Methods and Procedures*. A final sample of 30 people with multiple sclerosis. The outcome measures used were the ABILHAND questionnaire, the Purdue Pegboard Test, the Nine Hole Peg Test, and the Box and Block Test.

**Results:**

No significant correlations were found between dexterity and self-perception tests; however, correlations were found between perceived dexterity and quality of life (*p* < 0.001). Scores for the ABILHAND questionnaire, which measures the perception of skills in daily living, predicted up to 60% of the variance in the dexterity tests.

**Conclusions:**

The results of this study suggest that interventions for improving the manipulative dexterity of people with multiple sclerosis should address the person's perception of improving their manipulative dexterity and the perceived of quality of life, as both factors may influence manipulative dexterity.

## 1. Introduction

Multiple sclerosis (MS) is a multifocal, demyelinating disease that produces a progressive neurodegeneration of the central nervous system [1]. MS is classified into different subtypes: relapsing-remitting (RRMS), secondary progressive (SPMS), and primary progressive (PPMS). The disease affects young adults between 20 and 40 years, especially women (representing 2/3 of cases), and is the main cause of neurological nontraumatic disability in youth of developed countries. The estimated worldwide prevalence is 2.3 million people, and the incidence is 3-7 new cases per 100,000 habitants per year. In Spain, MS affects almost 50,000 people; this makes MS the second cause of acquired disability behind traffic accidents [1].

The MS affects functionality, interfering with the patient's ability to perform activities of daily living (ADLs), work activities, and leisure (regardless of subtypes, e.g., relapsing-remitting and progressive (primary and secondary)). As noted by De Groot et al. [2], at the onset of the disease, patients already present a significant functional decline. However, those with a progressive evolution from the appearance of the first symptoms present greater functionality difficulties and worse general health than those with the recurrent progressive and secondary progressive subtypes. This makes it difficult to predict the clinical course of the disease, which poses an important problem for clinicians treating patients with MS [2].

The impact of the disease is also reflected in the use of the upper limbs. It is estimated that three-quarters of patients with MS present had reduced manipulative dexterity, either unilaterally or bilaterally, which may also appear during initial stages of the disease [3, 4]. The disorders affecting the upper limbs caused by the disease due to sensory and motor problems, together with other disorders such as apraxia and fatigue, limit the person's ability to use the upper limb while performing ADLs [5–8]. This decrease in the manipulative capacity is related to an increased dependency, as well as having a negative impact on quality of life (QoL) [9]. Both the loss of fine motor skills and the decrease of hand strength are two of the most important predictive factors which decrease the ability to use the upper limbs in daily tasks, and this affects independence in performing ADLs [9–12]. In fact, the person's ability to perform daily activities which require the use of the upper limbs can be objectively and subjectively evaluated. Subjective tests are measured by questionnaires that assess the person's self-perception of perceived difficulties in performing tasks; in contrast, objective tests allow you to observe the person performing an activity which provides you with clearer information about the person's current and potential problems and captures the degree of third-person support needed to perform the activity. Both objective and subjective tests are important for a good assessment of the person's ADLs as they provide different points of view, but it should be noted that subjective tests provide a better understanding of the global nature of dexterity and are helpful for therapists to design rehabilitation interventions from a person-centered approach [13]. The ABILHAND [14] questionnaire evaluates the self-perceived ability to perform daily bimanual activities and is widely used in people with upper limb disability after a stroke, rheumatoid arthritis, systemic sclerosis, and MS and is a good observation tool to find out how the patient is feeling and how he/she perceives him/herself when performing the tasks [15–18].

The aims of this study were to analyze whether the manipulative dexterity disorders of people with MS are related to self-perceived QoL and performance and to explore self-perception influence on manipulative dexterity.

## 2. Methods

A cross-sectional descriptive study was performed in two associations of MS within the Community of Madrid, Spain. After obtaining signed informed consent, 35 patients with MS were recruited to participate in this study. The assessments were performed by five professionals, all of whom were occupational therapists.

This study was approved by the ethics committee of the University Rey Juan Carlos (registration's number: 220720153515). A cross-sectional descriptive study was performed in two associations of MS within the Community of Madrid, Spain. The study purpose and procedures were explained to the participants, and written informed consent was obtained in all cases. After obtaining signed informed consent, patients with MS were recruited to participate in this study. The assessments were performed by five professionals, all of whom were occupational therapists.

### 2.1. Participants

A sample of 35 patients from a rehabilitation center who attended consultations at the neurology services of their corresponding hospitals was recruited. Participants attending MS patient associations were asked to participate in the study. The inclusion criteria were individuals aged between 25 and 60 years, with a diagnosis of MS of the recurring-remitting subtype or the secondary progressive type in the same disease course, with an absence of relapses or flares in the last three months, and an absence of cognitive decline measured using the Montreal Cognitive Assessment (MoCA ≥ 26). The exclusion criteria included individuals with results higher than a score of 6 in the Expanded Disability Status Scale (EDSS) (requires constant assistance to walk) and/or those presenting previous depressive symptoms measured using the Beck Depression Inventory (BDI > 14, low depression); the higher the score, the greater the severity of depressive symptoms.

### 2.2. Assessment

#### 2.2.1. Manipulative Dexterity Assessment

The assessment of dexterity was performed using the following: (a) The Nine Hole Peg Test (NHPT) consists of placing nine pegs in their corresponding holes and then removing them as quickly as possible. The time the person takes to put in and remove the pins is recorded. It is performed with both hands; first, the time used by the dominant hand is measured and then that of the nondominant hand. The test is timed, and its duration depends on the patient's ability. The researchers have calculated population norms, and the time values contain both the expected mean number of seconds for the group and the standard deviation. NHPT has adequate reliability, and its validity has been confirmed in patients with multiple sclerosis [19, 20]. (b) The Box and Block Test (BBT) is easy and quick to administer (less than five minutes) and consists in moving the greatest number of wooden blocks (the test has 125 wooden blocks) from one compartment to another making them cross over a partition within 60 seconds. It has standardized instructions for administration and scoring. The subject must move, one by one, the maximum number of blocks from one compartment to another, starting with the dominant or unaffected hand and then with the nondominant or affected hand. The score obtained by each arm corresponds to the number of correctly displaced cubes. Higher scores are indicative of better manual dexterity [21]. Adequate reliability and validity of the BBT have been reported in the assessment of patients with neurological disorders, including multiple sclerosis [22]. (c) The Purdue Pegboard Test (PPT) consists of a board with holes and four cups with pins, collars, and washers. The participants must perform four subtests using the dominant hand, the nondominant hand, a bimanual task, and an assembly task. For each of these, subjects have 30 seconds except for assembly, where they are granted 60 minutes for the same. A higher pins, collars and washers put, higher manual dexterity. This test is easy and quick to administer (approximately 5 minutes' duration) [23]; also, it is a reliable assessment of manipulative dexterity in persons with multiple sclerosis [24]. (d) The ABILHAND questionnaire assesses the self-perceived capacity to perform bimanual daily activities. This questionnaire is easy to administer based on an interview and takes only 5-10 minutes. In total, 26 items are included with unimanual and bimanual activities. Each activity is scored on a three-level scale (0 = impossible, 1 = difficult, and 2 = easy). This shows that a higher score means a higher quality of life [14]. It is a reliable and valid method to assess patients with multiple sclerosis [25].

#### 2.2.2. Assessment of QoL

Quality of life was measured using two scales: (a) The European Quality of Life-5 Dimensions (EQ-5D), which consists of five descriptive questions which can be classified into five domains: mobility, personal care, activities of daily living, pain/discomfort, and anxiety/depression. Besides, it contains a graded scale from 0 to 100 (best imaginable health status) where patients must quantify their QoL on the present day [26]. Test-retest reliability and ceiling and floor effects of the EQ-5D demonstrated excellent reliability in persons with multiple sclerosis [27]. (b) The Multiple Sclerosis Quality of Life 54 (MSQoL-54) is a self-administered scale which is quick to administer (15-20 minutes). This test includes 18 specific items concerning MS, 54 in total. This assessment has a scale from 0 to 10 where subjects must grade their perception of quality of life in general [28]. The MSQOL-54 moderately detected change in health status and is fairly reliable and suitable for assessing the health-related quality of life of MS patients [29].

### 2.3. Statistical Analysis

First, descriptive data were obtained for the complete sample, in terms of levels of functionality, QoL, manipulative dexterity, and personal and sociodemographic variables. The frequency of the categorical variables was obtained, such as the mean and the median, and, in some cases, also for the continuous variables. Also, the homogeneity of the sample was measured to confirm that not all variables fulfilled the normality by histograms and was confirmed by the Shapiro-Wilk test. Therefore, parametric and nonparametric tests were used.

Subsequently, the possible correlations that existed between the different variables were assessed using Spearman's nonparametric test and Pearson's parametric test. Also, the influence of the examined factors in this study was examined using multivariate linear regressions. Multiple linear regression models were made and adjusted for the dexterity tools in order to determine the possible influence of motor dexterity and the physical and mental health composites of the quality of life of people with multiple sclerosis. The Durbin-Watson test was used for the regression analysis because it helps analyze the residuals for establishing the robustness among tested models.

The statistical analysis was performed using the IBM SPSS Statistics program for Windows, version 22.0 (Copyright © 2021 IBM SPSS Corp.).

## 3. Results

Of the total sample (*n* = 35 participants), 30 subjects eventually completed the study, of which there were 17 women and 13 men, with a mean age of 45.87 ± 8.11 years. Four people were excluded as they failed to fulfill the selection criteria, and one did not finish all the programmed assessments.

The left upper limb was the most affected in 56.7% of cases (*n* = 17), whereas the right upper limb was most affected in 43.3% of cases, and most of the sample were right-handed (93.3%).  Table 1 displays the descriptive data of the tests administered, as well as the personal and sociodemographic variables of the complete sample.

Significant positive correlations were found between the ABILHAND test and all the quality of life measures, except in the following dimensions of the MSQoL-54: energy (*p* = 0.205), social function (*p* = 0.136), global quality of life (*p* = 0.752), emotional wellbeing (*p* = 0.248), cognitive function (*p* = 0.346), and role limitations due to emotional problems (*p* = 0.61). This shows that a higher self-perceived capacity correlated with higher quality of life measures. In the tests for manipulative dexterity such as the NHPT, BBT, and PPT, no significant correlation was found either with the ABILHAND or with any quality of life scale, except in the performance of the bilateral PPT task, and for the total score, the scores of the PPT correlated (*r* = 0.532; *p* < 0.002; *r* = 0.413; *p* < 0.023) with the physical function item of the MSQoL-54 scale.  Table 2 displays all the significant correlations found in the complete sample.

With the intention of deepening our knowledge regarding the significant relation between the variables and the sample, an analysis of the variance of the perception tests was performed (MsQoL-54, EQ-5D, and ABILHAND) as possible predictors of the results found in the dexterity tests (PPT, NHPT, and BBT). The analysis was performed via linear regression, using all the items related to perception as predictive variables. The results revealed that the perception scales predict up to 60% of PPT variance, 19% of dominant BBT variance, 40% of nondominant BBT, 46% of dominant NHPT, and 33% of nondominant NHPT (Table 3).

## 4. Discussion

One of the aims of the present study was to provide a response to the possible relationship between the manipulative dexterity disorders and perceived performance and quality of life in people with MS.

As indicated by Johansson et al. [3], in MS, the presence of hand dysfunction and, therefore, of affected manual dexterity is evident even with low EDSS scores. Our study obtained data which detected a certain ability to perform activities successfully, with some difficulties appearing in the most difficult tasks, which usually correspond with bilateral tasks [4]. This is in line with other reports [30, 31], considering that the limitation of both upper limbs affects general functionality, and is one of the factors that presents a strong association with restrictions in participation and the perceived ability to perform everyday activities [32].

According to the findings of this study, there is no relationship between the self-perceived dexterity and the actual ability of a person with MS; rather, there is an association between self-perceived manual abilities and several dimensions of QoL. Nonetheless, we observed that the perception of the manipulative ability does not have a negative effect on some dimensions of QoL (energy, social function, global quality of life, emotional wellbeing, and cognitive function), perhaps because limitations only appear in more complex activities, and does not interfere with participation. The repercussion of the illness on QoL has been extensively documented. According to several studies, the progression of MS produces an increase in physical disability, which has a negative impact on physical aspects of the quality of life for people with MS [31, 32]. The individuals with MS perceive themselves as performing worse or less efficiently than before regarding certain daily activities; this will lead to either abandoning or performing these activities less often, which will unavoidably affect their quality of life. This corresponds with previous studies [33–35], which indicate how the state of mind of the person with MS affects their perceived health and quality of life.

Another aim of the present study consisted in exploring the influence of self-perception on manipulative dexterity. According to our findings, we have observed that self-perception of manual dexterity and quality of life involves areas of both physical and mental health, with a predictive capacity over manual dexterity. This finding may be due to the fact that the instrument used (MSQoL-54) is specially designed for MS [36, 37] and, therefore, is more sensitive and specific regarding changes affecting the disease in itself. A negative self-perception of dexterity and quality of life, either global or specific, produces a tendency for low scores on the dexterity scales, both gross and fine. This could be due to the positive influence of self-perception on motivational factors which involve an increased repetition of the task and involvement in activities of daily living which require a greater manipulative dexterity. If a person stays motivated, they will possibly try to maintain participation in activities; otherwise, it is quite likely that the person will abandon the same. As soon as the person abandons or performs these activities less often, a gradual loss of skill or dexterity caused by disuse may occur [34]. Other longitudinal studies [31, 38] conclude that, despite presenting greater physical disability and a progressive dependency in ADLs, people with mild MS maintain their participation in social activities over time as a result to maintain motivation. This coincides with our results, as the mean duration of the illness of subjects was 10 years. This suggests that, in order to deal with the psychological impact of the illness, people with MS develop appropriate coping and compensation strategies, including behavior changes and the modification of environmental factors, in order to maintain social participation.

Many clinical trials are available in the literature [8, 39, 40] in which objective tests are used to measure dexterity and their limitations. However, we highlight the importance of complementing this assessment by including the patient's perception of their manual dexterity. In this sense, the ABILHAND questionnaire (although not a specific instrument for MS) has proven to be valid when used with patients with MS [40, 41]. Marrie et al. [42] performed a study in which correlations were found between the ABILHAND questionnaire and other dexterity measurements such as the Performance Scales© in the domains of hand functionality, sensory assessments, and spasticity, concluding that the ABILHAND questionnaire may be a useful complementary assessment for the evaluation of the upper limb in MS. As we have observed in our study, the perception that a patient has on their dexterity predicts the objective results obtained via the manual dexterity tests. Therefore, we consider that the patient's self-perception of their manual dexterity provides valuable information which therefore should be included in the assessment of the manipulative capacity of people with MS.

The present study has a great limitation regarding the small sample size as this limits the possibilities of extracting generalizable conclusions. Future studies with greater samples are necessary to confirm these findings.

## 5. Conclusions

The findings of this study present clinical implications which are worth considering. The subjective perception of the person regarding their own dexterity in the performance of tasks is associated with their quality of life (physical and mental). However, although self-perception and manipulative dexterity skills are not objectively related in a specific moment in time, our data display that the subjective perception of the person is a predictive factor for manipulative dexterity in the progressive process of the disease. Therefore, based on the findings of this study, we suggest an intervention approach based on aspects related to the subjective perception of manipulative dexterity and quality of life of the person with MS due to the implications that this entails for manipulative dexterity skills.

## Figures and Tables

**Table 1:** Sample characteristics.

	*n* = 30
Age, mean ± SD (years)	45.87 ± 8.11 (31-58)
Sex, women (*n* (%))/men (*n* (%))	17 (56.7)/13 (43.3)
Educational level, basic/high-school/university (*n* (%))	4 (13.3)/14 (46.7)/12 (40)
Hand dominance, right (*n* (%))/left (*n* (%))	28 (93.3)/2 (6.7)
Most affected side, right (*n* (%))/left (*n* (%))	13 (43.3)/17 (56.7)
Years duration of the disease, mean ± SD	10.13 ± 7.06 (0.50-27)
EDSS, median IQR	6 (3.5-6)
MoCA, median IQR	26 (23-28)
PPT dominant, median IQR	9 (6-13)
PPT nondominant, median IQR	8.5 (6-11.25)
PPT bilateral, median IQR	6 (3-9)
PPT assembly, median IQR	17.5 (10-22.25)
PPT total, median IQR	23.5 (18-31.5)
NHPT dominant, median IQR	25.12 (2.04-35.38)
NHPT nondominant, median IQR	29.53 (23.95-35.4)
BBT dominant, median IQR	47 (34.75-52.75)
BBT nondominant, median IQR	44.5 (35.75-51.5)
ABILHAND, median IQR	1.9 (1.18-3.32)

BBT: Box and Block Test; EDDS: Expanded Disability Status Scale; IQR: interquartile range; MoCA: Montreal Cognitive Assessment; *N*: number of cases enrolled; NHPT: Nine Hole Peg Test; PPT: Purdue Pegboard Test; SD: standard deviation.

**Table 2:** Correlations among the different variables.

	EQ-5D	EQ-5D (perceived percentage)	MSQoL-54 (physical health)	MSQoL-54 (physical function)	MSQoL-54 (health perception)	MSQoL-54 (energy)	MSQoL-54 (lim. physical role)	MSQoL-54 (pain)	MSQoL-54 (physical health distress)	MSQoL-54 (mental health)	MSQoL-54 (mental health distress)
PPT bilateral	*ρ* = 0.059, 0.758	*r* = −0.120, 0.526	*r* = 0.106, 0.576	*r* = 0.532^∗∗^, 0.002	*ρ* = −0.107, 0.573	*ρ* = −0.134, 0.481	*ρ* = 0.227, 0.228	*ρ* = −0.156, 0.410	*r* = 0.152, 0.422	*r* = −0.192, 0.310	*r* = 0.133, 0.483
PPT total	*ρ* = 0.105, 0.580	*r* = −0.135, 0.475	*r* = 0.032, 0.866	*r* = 0.413^∗∗^, 0.023	*ρ* = −0.084, 0.660	*ρ* = −0.224, 0.234	*ρ* = 0.229, 0.224	*ρ* = −0.118, 0.535	*r* = 0.120, 0.528	*r* = −0.153, 0.420	*r* = 0.130, 0.493
NHPT nondominant	*ρ* = 0.137, 0.470	*r* = 0.089, 0.639	*r* = −0.036, 0.851	*r* = −0.317, 0.088	*ρ* = 0.210, 0.265	*ρ* = 0.361^∗^, 0.050	*ρ* = −0.075, 0.693	*ρ* = 0.251, 0.181	*r* = −0.153, 0.418	*r* = −0.087, 0.649	*r* = −0.144, 0.447
ABILHAND	*ρ* = 0.436^∗^, 0.016	*r* = 0.405^∗^, 0.026	*r* = 0.521^∗∗^, 0.003	*r* = 0.366^∗^, 0.046	*ρ* = 0.559^∗∗^, 0.001	*ρ* = 0.302, 0.105	*ρ* = 0.477^∗∗^, 0.008	*ρ* = 0.555^∗∗^, 0.001	*r* = 0.428^∗^, 0.018	*r* = 0.395^∗^, 0.031	*r* = 0.439^∗^, 0.015

^∗^Significant correlation with *p* < 0.05. ^∗∗^Significant correlation with *p* < 0.001. Reported statistics are *R* for Pearson correlations and *ρ* for Spearman correlations. EQ-5D: European Quality of Life-5 Dimensions; MSQoL-54: Multiple Sclerosis Quality of Life 54; NHPT: Nine Hole Peg Test; PPT: Purdue Pegboard Test.

**(a):** 

Predictive variables	Variance											
	Physical function		Limitation role physical		Pain		Physical health distress		Global QoL		ABILHAND	
	*β*	Sig	*β*	Sig	*β*	Sig	*β*	Sig	*β*	Sig	*β*	Sig
PPT (*R*^2^: .60; Durbin-Watson: 1.878)	0.787	0.050	0.816	0.033	-2.05	0.000	0.888	0.086	-1.458	0.016	2.313	0.020

**(b):** 

Predictive variables	Variance									
	MSQoL-54 physical health		Health perception		Pain		Emotional wellbeing		ABILHAND	
	*β*	Sig	*β*	Sig	*β*	Sig	*β*	Sig	*β*	Sig
NHPT dominant (*R*^2^: .45; Durbin-Watson: 1.828)	-1.5	0.048	10.05	0.009	9.46	0.011	-3.34	0.052	-15.11	0.009

**(c):** 

Predictive variables	Variance							
	MSQoL-54 physical health		Health perception		Pain		ABILHAND	
	*β*	Sig	*β*	Sig	*β*	Sig	*β*	Sig
NHPT nondominant (*R*^2^: .33; Durbin-Watson: 1.569)	-2.0	0.036	12.53	0.009	7.98	0.058	-14.33	0.040

**(d):** 

Predictive variables	Variance					
	Health perception		Limitation role physical		ABILHAND	
	*β*	Sig	*β*	Sig	*β*	Sig
BBT nondominant (*R*^2^: .40; Durbin-Watson: 2.491)	-3.1	0.002	1.059	0.050	4.12	0.020

**(e):** 

Predictive variables	Variance			
	Physical health distress		Mental health distress	
	*β*	Sig	*β*	Sig
BBT dominant (*R*^2^: .19; Durbin-Watson: 1.618)	-8.2	0.000	7.48	0.027

BBT: Box and Block Test; MSQoL-54: Multiple Sclerosis Quality of Life 54; NHPT: Nine Hole Peg Test; PPT: Purdue Pegboard Test; QoL: quality of life.

## Data Availability

The data used to support the findings of this study are included within the article. However, for more information, requests for access to these data should be made to Cristina García-Bravo (cristina.bravo@urjc.es).
